# *Porphyromonas gingivalis* under palmitate-induced obesogenic microenvironment modulates the inflammatory transcriptional signature of macrophage-like cells

**DOI:** 10.1371/journal.pone.0288009

**Published:** 2023-06-29

**Authors:** Xavier Minne, Joanna Mbuya Malaïka Mutombo, Fatiha Chandad, Roberto D. Fanganiello, Vanessa P. Houde

**Affiliations:** Faculty of Dentistry, Oral Ecology Research Group (GREB), Université Laval, Quebec City, Quebec, Canada; Yerevan State Medical University Named after Mkhitar Heratsi, ARMENIA

## Abstract

Metabolic diseases and low-grade chronic inflammation are interconnected: obese persons are at higher risk of developing periodontitis. However, the molecular mechanisms involved in the development and progression of periodontitis in an obesogenic microenvironment in response to periodontopathogens are still lacking. This study aims to investigate the combined effects of palmitate and *Porphyromonas gingivalis* on the secretion of pro-inflammatory cytokines and on transcriptional landscape modifications in macrophage-like cells. U937 macrophage-like cells were treated with palmitate and stimulated with *P*. *gingivalis* for 24h. Cytokines IL-1β, TNF-α and IL-6 were measured by ELISA in the culture medium and cell extracted RNA was submitted to a microarray analysis followed by Gene Ontology analyses. *P*. *gingivalis*, in presence of palmitate, potentiated IL-1β and TNF-α secretion in comparison to palmitate alone. Gene Ontology analyses also revealed that the combination palmitate-*P*. *gingivalis* potentiated the number of gene molecular functions implicated in the regulation of immune and inflammatory pathways compared to macrophages treated with palmitate alone. Our results provide the first comprehensive mapping of gene interconnections between palmitate and *P*. *gingivalis* during inflammatory responses in macrophage-like cells. These data highlight the importance of considering systemic conditions, specifically obesogenic microenvironment, in the management of periodontal disease in obese patients.

## Introduction

Periodontitis is an irreversible chronic disease induced by both the infiltration of immune cells into the gingival sulcus and the dysbiosis of the subgingival polymicrobial community. This dysbiotic state is thus impacting the host immune and inflammatory response in the gingival sulcus [1]. Untreated, periodontitis leads to destruction of the supporting tissues of the teeth, bone resorption and ultimately loss of teeth [2, 3]. Tissue destruction is caused by a highly inflamed environment linked to the production of inflammatory mediators, including cytokines such as interleukine-1β (IL-1β), tumor necrosis factor alpha (TNF-α) and interleukine-6 (IL-6) [4], matrix metalloproteinases (MMPs) [5], free radicals and oxidative stress molecules [6]. In the recent years, several studies have suggested that oral or systemic biomarkers could be used as tools for early detection of periodontitis. Among these biomarkers, Transforming growth factor-beta (TGF-β), Vascular endothelial growth factor (VEGF) and Receptor activator of NF-kB ligand (RANKL) were reported with promising results. In gingival fibroblasts, virulence factors of periodontopathogens, such as *Aggregatibacter actinomycetemcomitans* and *Porphyromomnas gingivalis*, are known to increase VEGF expression [7]. Moreover, in patients with periodontitis, TGF-β and VEGF are correlated with the evolution of the immune response [8]. Finally, high level of RANKL in gingival crevicular fluid has been associated with bone lost during periodontitis [9].

Obesity, an emerging worldwide epidemic condition, is defined as "an abnormal or excessive accumulation of fat that presents a risk to health” [10]. Adipose tissue is considered as an endocrine organ by itself where adipocytes, becoming larger, release pro-inflammatory mediators [11], which can influence the pathophysiology of several other health conditions [12]. This crucial role of adipose tissue in obesity-related systemic disease is linked to the accumulation of infiltrating macrophages into the adipose tissue and the subsequent secretion of pro-inflammatory cytokines by both macrophages and adipocytes [13]. Hence, obese persons are considered to be at higher risk of developing many chronic inflammatory diseases such as type 2 diabetes [14], cardiovascular diseases [15] and periodontitis [10, 16, 17]. In addition, periodontitis has been linked to, among other health conditions, obesity, type 2 diabetes, metabolic syndrome and cardiovascular diseases, revealing a bidirectional relationship between oral health and systemic health [18].

Obese and overweight individuals have high levels of circulating fatty acids, a condition known as hyperlipidemia [19]. Serum fatty acid level is known to increase by 26% in obese individuals compared to healthy subjects [20] and palmitate has been recognized as the predominant plasma saturated fatty acids in obese individuals [21]. Palmitate has been involved in the induction of inflammatory responses and it has been suggested that hyperlipidemia can be the key element linking obesity to periodontitis [22]. Moreover, macrophages can produce pro-inflammatory cytokines in response to palmitate-mediated activation of, among others, the Toll Like Receptor 4-Myeloid differentiation primary response 88-Nuclear factor-kappa B (TLR4-MyD88-NF-kB) signaling pathway [23]. Very few studies have addressed the impact of fatty acids or of an obesogenic microenvironment on periodontal inflammation at the molecular level. Some studies investigated the physiological effects of fatty acids on periodontal disease. For instance, alveolar bone loss has been observed in a rabbit model of periodontal disease fed with a high fat diet [24] and palmitate has also been associated with an increased inflammatory response in periodontal ligament fibroblasts subjected to mechanical forces and stimulated with lipopolysaccharides from *P*. *gingivalis* [25]. Nevertheless, the understanding of the molecular mechanisms involved in the development and progression of periodontal disease in an obesogenic microenvironment in response to periodontopathogens or their virulence factors is still scarce.

Palmitate and *P*. *gingivalis*, when studied individually, are known triggers of inflammation in immune cells [23, 26]. However, results on the impacts of their combination on molecular mechanism regulating inflammation are scarce. In the present study, we investigated the combined effects palmitate and *P*. *gingivalis* on the secretion of pro-inflammatory cytokines and on transcriptional landscape modifications in U937 macrophage-like cells. We found that the combination palmitate-*P*. *gingivalis* potentiates IL-1β and TNF-α secretion, is linked to a specific transcriptomic signature and modifies the expression of genes implicated in the regulation of immune and inflammatory pathways. Our data thus provide the first comprehensive mapping of the interconnections between palmitate and *P*. *gingivalis* during an inflammatory response.

## Materials and methods

### Materials

All chemicals were purchased from Sigma-Aldrich (Oakville, ON, Canada). Bacteria culture medium was purchased from Fisher Scientific (Ottawa, ON, Canada). U937 cell culture medium and heat-inactivated fetal bovine serum (HI-FBS) were purchased from Corning (Corning, NY, USA). RNA extraction kit was acquired from Zymo Research (Irvine, CA, USA). Duoset interleukine-1β (IL-1β), tumor necrosis factor alpha (TNF-α) and interleukine-6 (IL-6) ELISA kits were purchased from R&D Systems (Toronto, ON, Canada).

### Palmitate solution

We used palmitate to induce an obesogenic microenvironment in our cell culture system. Sodium palmitate was conjugated to fatty acid free (FFA) bovine serum albumin (BSA) and then administrated to the cells accordingly to the protocol from Xiu et al. [27]. Briefly, a 100 mM palmitate stock solution was made in 50% ethanol and heated at 70°C to allow the palmitate dissolution. A 5 mM working solution was then be prepared by diluting the 100 mM palmitate stock solution in a 5% FFA-BSA solution in cell culture medium. The 5 mM working solution was heated at 55°C for 10 minutes and mixed frequently. A 5% FFA-BSA solution in cell culture medium was used as vehicle for the cell treatments. The solutions were filtered through a 0.2 μm filter, aliquoted and store at -80°C for up to 3 months.

### Bacteria and growth conditions

*P*. *gingivalis* (ATCC 33277, Manassas, VA, USA) was grown in Todd-Hewitt Broth (THB) supplemented with 0.001% hemin and 0.0001% vitamin K at 37°C in anaerobic conditions (80% N_2_, 10% CO_2_, 10% H_2_). *P*. *gingivalis* was heat-inactivated for 30 minutes at 70°C to prevent the degradation of secreted pro-inflammatory cytokines in cell culture supernatant by *P*. *gingivalis’* proteases [28].

### U937 macrophage-like cells

Human U937 monocytes (ATCC CRL-1593.2) [29] were cultivated in Roswell Park Memorial Institute (RPMI) medium supplemented with 10% HI-FBS and 100 ug/ml penicillin/streptomycin + 0.25 ug/ml amphotericin B at 37°C in 5% CO_2_. 1 x 10^6^ cells/wells were seeded in 12-wells plate and monocytes were differentiated in macrophage-like cells with 50 ng/ml of phorbol 12-myristate 13-acetate (PMA) for 72h. Macrophage-like cells were then washed to remove non-adherence cells and incubated in RPMI supplemented with 1% HI-FBS for 24h prior the treatments. U937 macrophage-like cells were treated with 0.3 mM palmitate and/or stimulated with heat-inactivated *P*. *gingivalis* at a (multiplicity of infection) MOI of 10 (10 bacteria for 1 eukaryotic cell). Cells treated with the vehicle were used as controls. After 24h of incubation at 37°C in 5% CO_2_, the cell culture supernatant was collected and stored at -20°C.

### Pro-inflammatory cytokines secretion profile

ELISA kits were used to determine IL-6, IL-1β, and TNF-α concentrations in the cell culture supernatants accordingly to the manufacturer’s protocols (R&D Systems.) The cells were lysed, and the proteins quantified to normalize the results. Experiments were performed in 3 independent replicates and in technical triplicate for each condition. Results were analyzed using two-way ANOVA test in combination with a Tukey *post hoc* test to assign statistical significance (p < 0.05).

### Transcriptional landscape analyses

U937 macrophage-like cells were treated with 0.3 mM palmitate and stimulated with heat-inactivated *P*. *gingivalis* at a MOI of 10. Cells treated with the vehicle were used as controls. After 24h of incubation at 37°C in 5% CO_2_, the cell culture supernatant was removed, and cells were washed with ice-cold PBS. RNA was then extracted using the extraction kit from the manufacturer’s protocols (Zymo Research). The concentration of RNA was determined with a Nanodrop device (ThermoFisher, Mississauga, ON, Canada). 150 ng of RNA was submitted to gene microarray (Pico Clariom S, Affymetrix, Santa Clara, CA, USA) and sequenced by Centre d’expertise et de service Génome Québec (Montreal, QC, Canada). Experiments were performed twice in single biological replicates and the results were analyzed with the Transcriptome Analysis Console (TAC) Software version 4.0.2.15 from Affymetrix. Transcripts with False Discovery Rate (FDR) p-value < 0.05 and absolute log2 foldchanges > 1.8 were considered. The microarray data were deposited in the BioStudies database server (https://www.ebi.ac.uk/biostudies/studies) under accession number: S-BSST910. A Venn diagram was generated with the Bioinformatics & Evolutionary Genomics online tool from the University of Gent (available online at https://bioinformatics.psb.ugent.be/webtools/Venn). To gain insights into the gene expression signatures, Gene Ontology analyses were performed by using ShinyGO, a bioinformatic tool allowing simultaneous visualization of enrichment results, statistical analyses and interface access to KEGG and STRING [30] (available online at http://bioinformatics.sdstate.edu/go/, Edge cut-off = 0.3 and FDR p-value cut-off = 0.05).

## Results

### Palmitate potentiates IL-1β and TNF-α secretion by macrophage-like cells in presence of the periodontopathogen *P*. *gingivalis*

**Fig 1:**
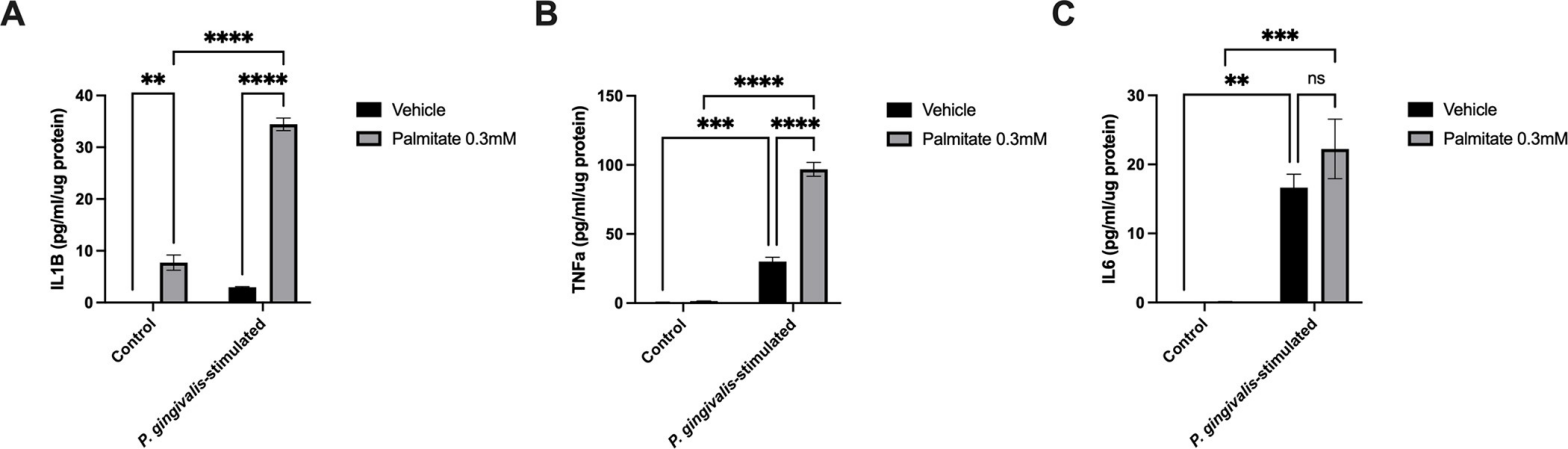
Palmitate potentiates IL-1β and TNF-α secretion by U937 macrophage-like cells in presence of the periodontopathogen *P*. *gingivalis*. Cells were either treated with the vehicle or palmitate 0.3 mM and stimulated or not with *P*. *gingivalis* for 24 hours. (**A)** IL-1β, (**B)** TNF-α and (**C)** IL-6 were measured by ELISA in the cell culture supernatant. Two-Way ANOVA with Tukey *post hoc* test was performed, and results are the means ± SEM. ** p < 0.01, *** p < 0.001 and **** p < 0.0001. n = 3 (three) independent experiments in triplicate.

To assess the effect of palmitate and *P*. *gingivalis* on the secretion of pro-inflammatory cytokines, we measured the accumulation of IL-1β, TNF-α and IL-6, three hallmark molecules associated with gingival inflammation [4], in the culture media of U937 macrophage-like cells. Treatment with 0.3 mM palmitate for 24h induced the secretion of IL-1β (Fig 1A) by a 59-fold increase but not of TNF-α nor IL-6 (Fig 1B, 1C) compared to untreated cells. Treatment of cells with 0.3 mM palmitate and stimulation with *P*. *gingivalis* potentiated IL-1β and TNF-α secretion leading to 4.5-fold and 64-fold increases, respectively, in their production in comparison to the cells treated with the palmitate alone (Fig 1A, 1B). Stimulation with *P*. *gingivalis* alone increased the secretion of IL-1β, TNF-α and IL-6 by 22-fold, 50-fold and 491-fold respectively, in comparison to untreated cells (Fig 1A–1C).

### Palmitate, *P*. *gingivalis* and the combination of both engender three specific gene expression signatures in macrophage-like cells

**Fig 2:**
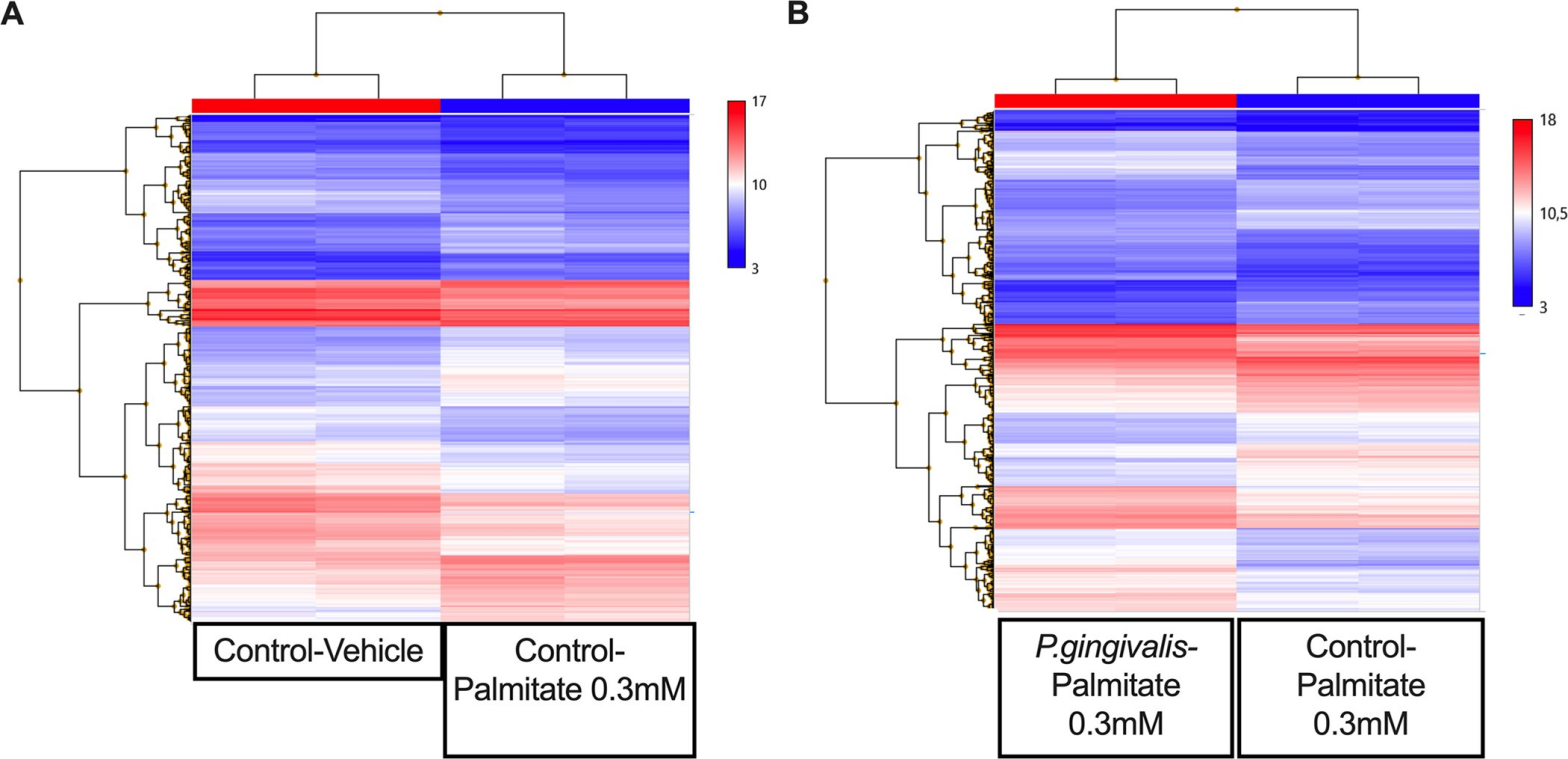
Hierarchical cluster of gene expression of U937 macrophage-like cells. U937 macrophage-like cells were treated with (**A)** the vehicle (Control-Vehicle) compared to cells treated with palmitate (Control-Palmitate 0.3 mM) for 24h and (**B)** U937 macrophage-like cells treated with palmitate 0.3 mM (Control-Palmitate 0.3 mM) compared to cells treated with palmitate and stimulated with the periodontopathogen *P*. *gingivalis* (*P*. *gingivalis*-Palmitate 0. 3mM) for 24h. Microarray analysis was performed using the Affymetrix Clariom S Pico Array. 1.8-fold upregulated and downregulated genes were clustered with the Affymetrix Transcription Analysis Console (TAC) software (v.4.0.2.15). Each array was performed in duplicate.

To delve into transcriptional landscape modifications linked to palmitate-induced obesogenic microenvironment and to investigate the impacts of *P*. *gingivalis* on inflammation, RNA extracted from U937 macrophage-like cells was submitted to microarray analysis. Principal component analysis (PCA) indicated that the variance in gene expression profiles differed between the experimental conditions and was similar between the biological replicates (S1 Fig). Out of a total of 21,448 transcripts analyzed, 596 transcripts were found to be differentially expressed (278 upregulated and 318 downregulated) in cells treated with palmitate when compared with the vehicle-treated cells, showing that palmitate generates a specific gene expression signature. Cell stimulation with *P*. *gingivalis* also prompts a distinct gene expression signature when compared with vehicle-treated cells, comprising 1214 differentially expressed genes (603 upregulated and 611 downregulated). These transcriptional landscapes change to a transcriptional signature consisting of 1185 transcripts (535 upregulated and 650 downregulated) when cells were treated with the combination *P*. *gingivalis*-palmitate compared to non-stimulated cells under the same environment. The differentially expressed transcripts in presence or in absence of palmitate and/or *P*. *gingivalis* are depicted by differences in heatmaps’ hierarchical clustering (Fig 2A, 2B and S2 Fig). Finally, a Venn diagram was generated to address overlapping in differentially expressed transcripts between these three gene expression signatures. We found that 154 transcripts were shared between these experimental conditions (S3 Fig).

### Macrophage-like cells under palmitate-induced obesogenic microenvironment treated with *P*. *gingivalis* display enrichment of molecular functions linked to the regulation of immune and inflammatory pathways

**Fig 3:**
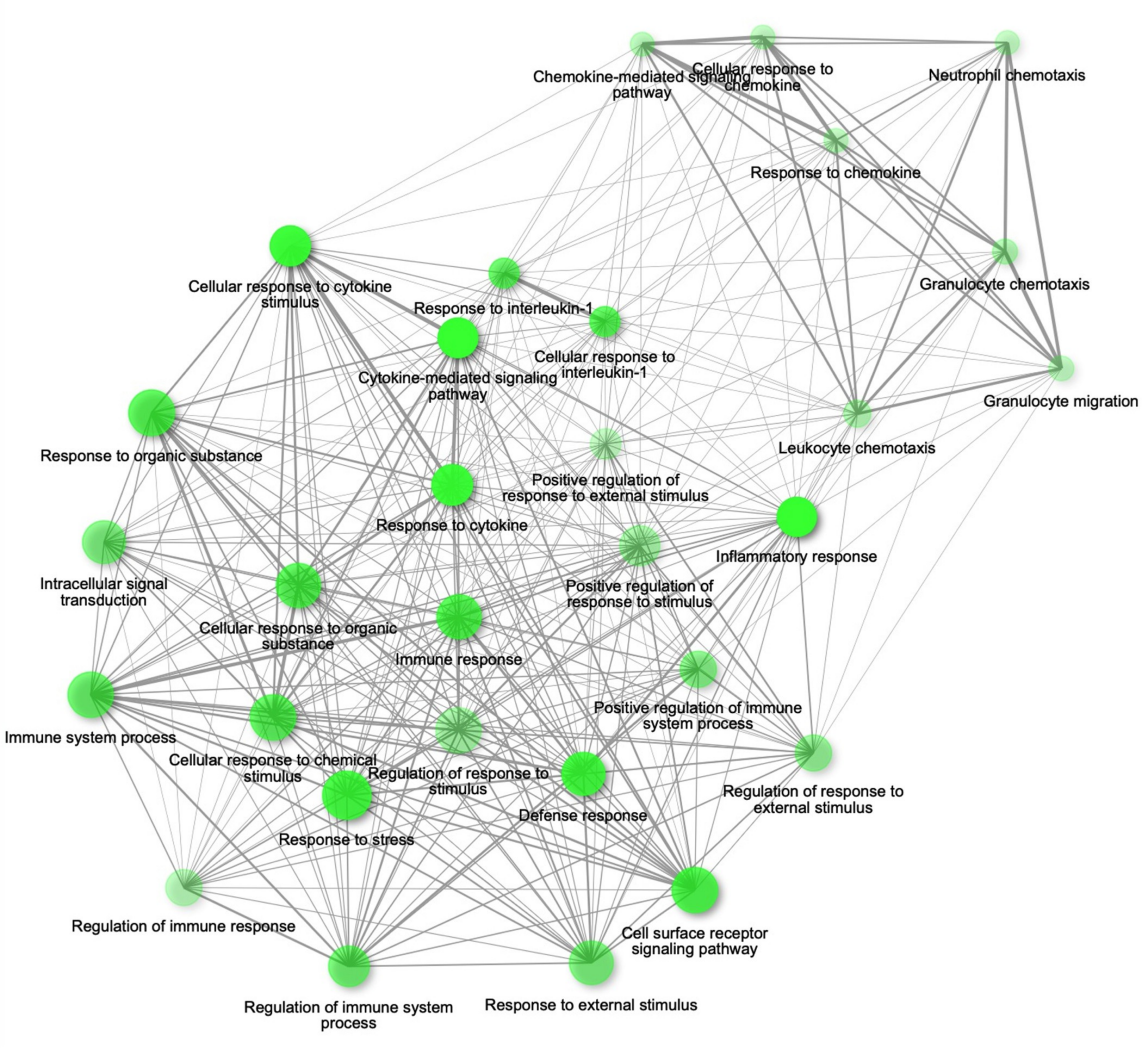
Relation between enriched biological processes of U937 macrophage-like cells treated with the vehicle (Control-Vehicle) compared to cells treated with palmitate (Control-Palmitate 0.3 mM) for 24h. The Gene Ontology analysis was performed using ShinyGo version 0.75 (http://bioinformatics.sdstate.edu/go/) that was accessed online on March 11^th^, 2022. Bigger nodes in bright green represent enriched biological processes while smaller nodes in opaque green represent smaller gene sets and less enriched biological processes, respectively. Highly overlapping processes are depicted by thicker edges.

**Fig 4:**
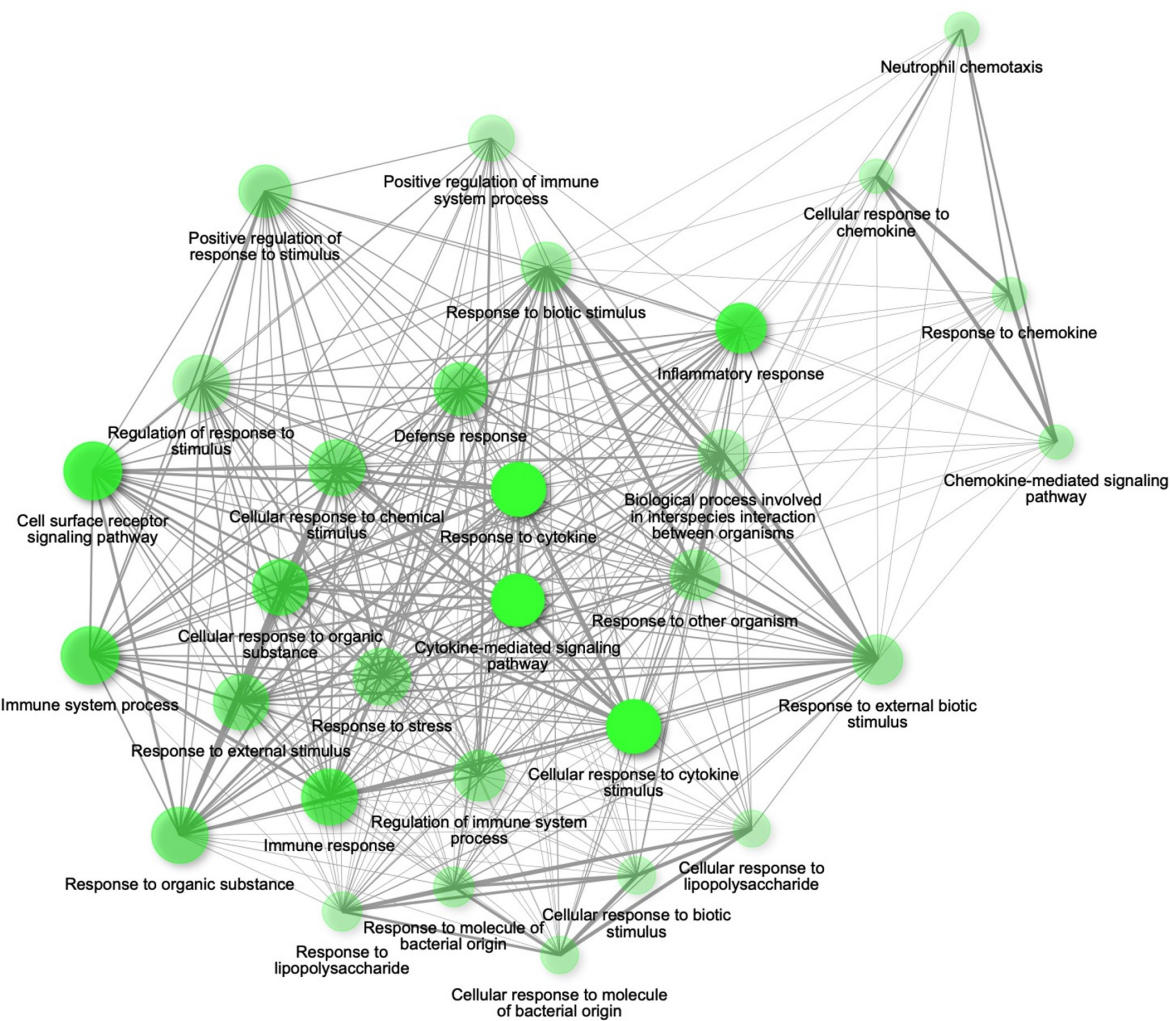
Relation between enriched biological processes of U937 macrophage-like cells treated with palmitate (Control-Palmitate 0.3 mM) compared to cells treated with palmitate and stimulated with the periodontopathogen *P*. *gingivalis* (*P*. *gingivalis*-Palmitate 0.3 mM) for 24h. The Gene Ontology was performed using ShinyGo version 0.75 (http://bioinformatics.sdstate.edu/go/) that was accessed online on March 11^th^, 2022. Bigger nodes in bright green represent enriched biological processes while smaller nodes in opaque green represent smaller gene sets and less enriched biological processes, respectively. Highly overlapping genes are depicted by thicker edges.

To further understand the interactions between the differentially expressed transcripts, we performed Gene Ontology analyses. First, a Gene Ontology analysis at the biological processes level by using the STRING database through ShinyGO was completed for the 154 shared transcripts identified with the Venn diagram. We found that these shared transcripts are implicated in, among other, biological processes, immune system process, inflammatory response, response to lipopolysaccharide and response to organic substance (S1 Table). Second, we performed Gene Ontology analyses with ShinyGO to characterize the processes and pathways that the differentially expressed transcripts regulate. We observed enrichment of inflammatory biological processes in *P*. *gingivalis*-stimulated cells (S4 Fig). Interestingly, palmitate induced enrichment of inflammatory pathways in which two major clusters were observed: 1) cytokines/inflammatory response/immune response and 2) chemokines (Fig 3). Moreover, a potentiation of this enrichment in inflammatory process was observed, as reflected by darker/larger nodes and thicker edges, after the treatment of cells with the combination *P*. *gingivalis*-palmitate (Fig 4) when compared to palmitate-treated cells (Fig 3).

Moreover, we performed Gene Ontology analyses at the molecular functions level by using the STRING database through ShinyGO. Treatment of cells with palmitate revealed activation of molecular functions that are responsive to immune and cytokine receptor activities as well as to signaling receptor binding and protein binding (Table 1) while stimulation with *P*. *gingivalis* led to an enrichment of cytokines molecular functions (S2 Table). Treatment of the cells with the combination palmitate-*P*. *gingivalis* significantly increased the number of molecular functions implicated in the regulation of immune and inflammatory pathways in comparison to the cells treated with palmitate (Table 2). These molecular functions were predominantly linked to interleukine-1 receptor binding, cytokine receptor binding, cytokine activity, signaling receptor binding, and protein binding.

**Table 1:** Representative enriched molecular functions of U937 macrophage-like cells treated with palmitate (Control-Palmitate 0.3 mM) compared to cells treated with the vehicle (Control-Vehicle) for 24h.

FDR	Number of genes	GO pathways	Description
0.0000	11	GO:0140375	Immune receptor activity
0.0000	9	GO:0004896	Cytokine receptor activity
0.0022	18	GO:0005102	Signaling receptor binding
0.0130	40	GO:0005515	Protein binding

The Gene Ontology was performed using ShinyGo version 0.75 (http://bioinformatics.sdstate.edu/go/) that was accessed online on March 11^th^, 2022.

**Table 2:** Representative enriched molecular functions of U937 macrophage-like cells treated with palmitate and stimulated with the periodontopathogen *P*. *gingivalis* (*P*. *gingivalis*-Palmitate 0.3 mM) compared to cells treated with palmitate (Control-Palmitate 0.3 mM) for 24h.

FDR	Number of genes	GO pathways	Description
0.0000	26	GO:0005126	Cytokine receptor binding
0.0000	22	GO:0005125	Cytokine activity
0.0000	38	GO:0005102	Signaling receptor binding
0.0000	68	GO:0005515	Protein binding
0.0004	4	GO:0005149	Interleukine-1 receptor binding
0.0007	5	GO:0032813	Tumor necrosis factor receptor superfamily binding
0.0013	6	GO:0004222	Metalloendopeptidase activity
0.0042	3	GO:0035325	Toll-like receptor binding

The Gene Ontology was performed using ShinyGo version 0.75 (http://bioinformatics.sdstate.edu/go/) that was accessed online on March 11^th^, 2022.

## Discussion

Microorganisms and their specific virulence factors, such as lipopolysaccharide (LPS), peptidoglycans and lipoteichoic acid, are important inducers of inflammation [31]. In active periodontal lesions, key players regulating the host inflammatory response are the monocytes and macrophages through the infiltration of the gingival connective tissues [32]. Therefore, monocytes/macrophages are crucial modulators of the initiation and progression of periodontal disease by secreting pro-inflammatory cytokines and matrix metalloproteinases (MMPs) [33, 34] as well as generating reactive oxygen species (ROS) [35]. Inflammation and metabolic diseases, such as obesity, are interconnected and a dysfunction of this bidirectional system impacts systemic health [12]. Recently, it has been recognized that obesity affects the periodontium ecosystem, particularly periodontal inflammation, in a reciprocal manner [10, 36]. Obese individuals have thus a higher risk of onset or progression of periodontal disease [16, 37–40]. Many mechanisms have been proposed to explain this relationship. They are most likely linked to the regulation of the immune response through the secretion of pro-inflammatory cytokines by macrophages infiltrating both the expended adipose tissues and the gingival sulcus [10, 11, 16]. In addition, hyperlipidemia is known to blunt the immune response to the periodontopathogen *P*. *gingivalis* in apolipoprotein E knockout mice [41]. Alveolar bone loss has also been associated with obesity in a mouse model of *P*.*gingivalis*–induced periodontal disease fed with a high fat diet [42]. In the present study, we found that U937 macrophage-like cells treated with the combination *P*. *gingivalis*-palmitate display specific gene signatures and enrichment of molecular functions linked to the regulation of immune and inflammatory pathways.

Several potential inflammatory triggers have been described in relation to chronic inflammation induced by fatty acids or periodontopathogens. However, the combined impacts of palmitate and *P*. *gingivalis* exposure are very scarce. We speculate that the exposure of U937 macrophage-like cells to *P*. *gingivalis*-palmitate combination may affect mitochondrial functions. It has been demonstrated in *in vitro* studies that palmitic acid increased reactive oxygen species (ROS) production by inhibiting the complexes I and complexes III of the mitochondrial respiratory chain in immune cells [43, 44]. Furthermore, palmitate overload has been shown to downregulate genes regulating mitochondrial oxidative phosphorylation [45]. In addition, *P*. *gingivalis* lipopolysaccharides can induce mitochondrial dysfunction by reducing mitochondrial membrane potential and mitochondrial protein expression [46, 47]. Further studies are under investigation to confirm the involvement of *P*. *gingivalis*-palmitate combination in the regulation of mitochondrial functions.

Moreover, Toll-like receptor 4 (TLR4) is activated by circulating fatty acids [48] resulting in secretion of pro-inflammatory cytokines [23, 49]. It has been reported that palmitate augmented IL-6 secretion by primary human gingival cells and human periodontal ligament fibroblasts [25, 50]. In contrast to this finding, we did not observe an increase in IL-6 secretion by cells treated with palmitate. In light of this finding, we propose that immune and non-immune cells do not use the same signaling pathways to control pro-inflammatory cytokines secretion in response to palmitate. Our results revealed that palmitate strongly induced IL-1β secretion. In addition, we have demonstrated a potentiation of IL-1β and TNF-α secretion by the macrophages treated with palmitate and stimulated with *P*. *gingivalis*. These data suggest a specificity of palmitate, in combination with *P*. *gingivalis*, on regulating the secretion of the superfamily of interleukine-1 cytokines in U937 macrophage-like cells. The mechanisms behind this potentiation of IL-1β secretion in presence of the combination palmitate-*P*. *gingivalis* is under investigation.

At of the time of this writing our study is the first one to delve into and unravel interconnections in gene expression circuitry between inflammation, palmitate, and *P*. *gingivalis*. It has been demonstrated that the combination palmitate-*P*. *gingivalis* increased palmitate-induced chemokine secretion in primary human gingival cell culture supernatants [50]. Our study also reported a potentiation of palmitate-induced chemokine genes expression supporting the fact that palmitate stimulates a chemokine response. In addition, our results showed increased expression of genes involved in Toll-like receptor, pro-inflammatory cytokines, and matrix metalloproteinases expression suggesting a very intricate inflammatory interplay between palmitate and *P*. *gingivalis*. Furthermore, the Gene Ontology analysis allowed a comprehensive visualization of these complex inflammatory interconnections. Our results revealed an increase in the number of gene molecular functions implicated in the regulation of immune and inflammatory pathways in cells treated with the combination palmitate-*P*. *gingivalis* when compared with palmitate alone, suggesting that the same signaling pathways are most likely activated, in a synergistic manner, potentiating the inflammatory response. We also speculate that *P*. *gingivalis* can dysregulate lipid signaling pathways in obesogenic environment as previously described for oleic acid in an immortalized liver cell line [51]. Given that inflammation is most likely the key factor linking periodontitis and obesity, it is important to identify inflammatory markers that can be used for early diagnosis of periodontitis in obese individuals. Recently, several oral and systemic common biomarkers have been described. Elevated level of the C-reactive protein (CRP) has been reported in obese patients with periodontitis [52] while overexpression of VEGF has been observed in inflamed periodontal tissues of patients with type 2 diabetes, another chronic inflammatory disease often associated with obesity [53]. Although we did not study modifications of VEGF and CRP expression in our transcriptional landscape experiment, we can hypothesize that the transcriptional signature observed in our experimental model can be used to identify early common biomarkers for early diagnosis and management of periodontitis in obese individuals. However, further studies should be performed to directly investigate this hypothesis.

Dietary inflammation can be associated with periodontal health [18, 54] and a bidirectional relationship between nutrition, dietary intake and oral health has been recently recognized. For example, saturated fat-rich or hypercaloric diets can induce oxidative stress or inflammation and should be avoided to prevent periodontitis or to achieve better results after periodontal therapies [55]. Woelber also reported that a low-carbohydrate, rich-Omega 3 fatty acids diet can reduce gingival and periodontal inflammation [56]. However, the role of individual nutrients in inducing inflammation is still unclear [54]. Nutrition and dietary intake should thus be considered to improve the clinical outcomes during periodontal therapies in both obese and non-obese individuals. Large scale controlled randomized dietary-interventional studies are needed to evaluate this hypothesis.

## Conclusion

Obesity and periodontitis are two chronic inflammatory diseases linked by underlying biological mechanisms, in which inflammation plays a key role. However, very few studies have, to date, addressed the combined effects of palmitate and the periodontopathogen *P*. *gingivalis* on gingival inflammation at the molecular level. Our studies provide the first comprehensive mapping of gene interconnections responsible for the regulation of inflammatory responses between palmitate and *P*. *gingivalis* in human U937 macrophage-like cells which, in turn, resulted in a potentiation of IL-1β and TNF-α secretion. These data reveal the potential of obesogenic markers in increase of destructive process during periodontitis. These data also highlight the importance of considering systemic conditions, specifically obesogenic microenvironment in the management of periodontal disease in link with obesity.

## Supporting information

S1 Table Representative enriched biological processes of U937 macrophage-like cells treated with the vehicle (Control-Vehicle), stimulated with the periodontopathogen *P*. *gingivalis* (*P*. *gingivalis*-Vehicle) and treated with the combination *P*. *gingivalis*-palmitate (*P*. *gingivalis*-Palmitate 0.3mM) for 24h. (DOCX)

S2 Table Representative enriched molecular functions of U937 macrophage-like cells treated with the vehicle (Control-Vehicle) compared to cells stimulated with the periodontopathogen *P*. *gingivalis* (*P*. *gingivalis*-Vehicle) for 24h. (DOCX)

S1 Fig Differences in transcriptome profiles following treatment with palmitate and stimulation with the periodontopathogen *P*. *gingivalis*. The graph depicts a 3D plot of the first 3 principal component analysis (PCA) of U937 macrophage-like cells normalized microarray data. The Affymetrix Transcription Analysis Console (TAC) software (v.4.0.2.15) was used to perform the PCA analysis. Each array was performed in duplicate. The blue dots represent the Control-Vehicle cells (CTL), the red dots represent the Control-Palmitate 0.3mM cells (Palm), the purple dots represent the *P*. *gingivalis*-Vehicle cells (CTL Pg) and the green dots represent the *P*. *gingivalis*-Palmitate 0.3mM cells (Palm Pg).
(PDF)

S2 Fig Hierarchical cluster of gene expression of U937 macrophage-like cells treated with the vehicle (Control-Vehicle) compared to cells stimulated with the periodontopathogen *P*. *gingivalis* (*P*. *gingivalis*-Vehicle) for 24h. Microarray analysis was performed using the Affymetrix Clariom S Pico Array. 1.8-fold upregulated and downregulated genes were clustered with the Affymetrix Transcription Analysis Console (TAC) software (v.4.0.2.15). Each array was performed in duplicate.
(PDF)

S3 Fig Venn diagram showing overlap of differentially expressed transcripts in U937 macrophage-like cells treated with palmitate (Control-Palmitate 0.3mM), treated with the periodontopathogen *P*. *gingivalis* (*P*. *gingivalis*-Vehicle) and treated with the combination *P*. *gingivalis*-palmitate (*P*. *gingivalis*-Palmitate 0.3mM) for 24h. Microarray analysis was performed using the Affymetrix Clariom S Pico Array. 1.8-fold upregulated and downregulated genes were clustered with the Affymetrix Transcription Analysis Console (TAC) software (v.4.0.2.15). The Venn diagram was generated with the Bioinformatics & Evolutionary Genomics online tool from the University of Gent (available online at https://bioinformatics.psb.ugent.be/webtools/Venn). Each array was performed in duplicate.
(PDF)

S4 Fig Relation between enriched biological processes of U937 macrophage-like cells treated with the vehicle (Control-Vehicle) compared to cells stimulated with the periodontopathogen *P*. *gingivalis* (*P*. *gingivalis*-Vehicle) for 24h. The gene ontology analysis was performed using ShinyGo version 0.75 (http://bioinformatics.sdstate.edu/go/) that was accessed online on March 11^th^, 2022. Bigger nodes in bright green represent enriched biological processes while smaller nodes in opaque green represent smaller gene sets and less enriched biological processes, respectively.
(PDF)
