# Supplementary material for: Porphyromonas gingivalis under palmitate-induced obesogenic microenvironment modulates the inflammatory transcriptional signature of macrophage-like cells
Source: PLoS One. 2023 Jun 29;18(6):e0288009. doi: 10.1371/journal.pone.0288009 (PMC10309636; doi:10.1371/journal.pone.0288009)
Supplement: S1 Table — (DOCX) [file pone.0288009.s001.docx]

| FDR | Number of genes | GO pathways | Description |
| --- | --- | --- | --- |
| 0.0000 | 53 | GO:0002376 | Immune system process |
| 0.0000 | 40 | GO:0002682 | Regulation of immune system process |
| 0.0000 | 22 | GO:0006954 | Inflammatory response |
| 0.0000 | 17 | GO:0032496 | Response to lipopolysaccharide |
| 0.0000 | 51 | GO:0010033 | Response to organic substance |
| 0.0000 | 25 | GO:0050776 | Regulation of immune response |
| 0.0000 | 24 | GO:0033993 | Response to lipid |
| 0.0000 | 21 | GO:0009617 | Response to bacterium |
| 0.0000 | 26 | GO:0045321 | Leukocyte activation |
| 0.0000 | 87 | GO:0050896 | Response to stimulus |

A Venn diagram was generated with the Bioinformatics & Evolutionary Genomics online tool from the University of Gent (available online at <https://bioinformatics.psb.ugent.be/webtools/Venn>). A Gene Ontology of the shared transcripts was then performed using ShinyGo (version 0.75 (<http://bioinformatics.sdstate.edu/go/>) that was accessed online on March 11^th^, 2022).
