# Supplementary material for: Porphyromonas gingivalis under palmitate-induced obesogenic microenvironment modulates the inflammatory transcriptional signature of macrophage-like cells
Source: PLoS One. 2023 Jun 29;18(6):e0288009. doi: 10.1371/journal.pone.0288009 (PMC10309636; doi:10.1371/journal.pone.0288009)
Supplement: S2 Table — (DOCX) [file pone.0288009.s002.docx]

| FDR | Number of genes | GO pathways | Description |
| --- | --- | --- | --- |
| 0.0000 | 17 | GO:0005126 | Cytokine receptor binding |
| 0.0000 | 15 | GO:0005125 | Cytokine activity |
| 0.0000 | 20 | GO:0005102 | Signaling receptor binding |
| 0.0000 | 6 | GO:0008009 | Chemokine activity |
| 0.0000 | 7 | GO:0004222 | Metalloendopeptidase activity |
| 0.0000 | 4 | GO:0005149 | Interleukine-1 receptor binding |
| 0.0004 | 5 | GO:0004896 | Cytokine receptor activity |

The Gene Ontology was performed using ShinyGo version 0.75 (<http://bioinformatics.sdstate.edu/go/>) that was accessed online on March 11^th^, 2022.
