# Supplementary figures and images for: Porphyromonas gingivalis under palmitate-induced obesogenic microenvironment modulates the inflammatory transcriptional signature of macrophage-like cells
Source: PLoS One. 2023 Jun 29;18(6):e0288009. doi: 10.1371/journal.pone.0288009 (PMC10309636; doi:10.1371/journal.pone.0288009)

# PCA Mapping 80,9% (CHP)

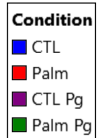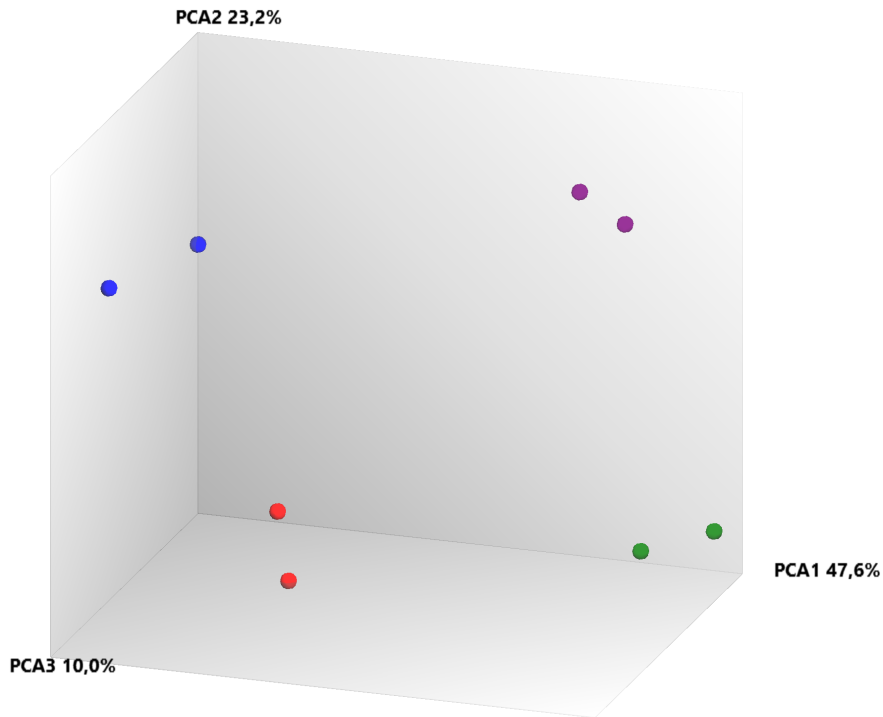

Supplement: S1 Fig — The graph depicts a 3D plot of the first 3 principal component analysis (PCA) of U937 macrophage-like cells normalized microarray data. The Affymetrix Transcription Analysis Console (TAC) software (v.4.0.2.15) was used to perform the PCA analysis. Each array was performed in duplicate. The blue dots represent the Control-Vehicle cells (CTL), the red dots represent the Control-Palmitate 0.3mM cells (Palm), the purple dots represent the P. gingivalis-Vehicle cells (CTL Pg) and the green dots represent the P. gingivalis-Palmitate 0.3mM cells (Palm Pg). (PDF) [file pone.0288009.s003.pdf]

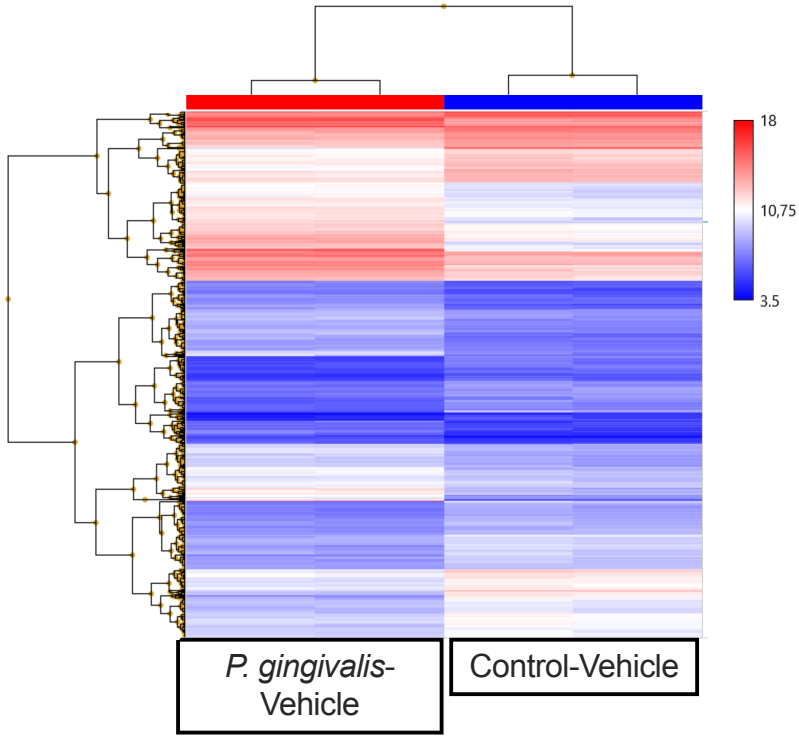

Supplement: S2 Fig — Microarray analysis was performed using the Affymetrix Clariom S Pico Array. 1.8-fold upregulated and downregulated genes were clustered with the Affymetrix Transcription Analysis Console (TAC) software (v.4.0.2.15). Each array was performed in duplicate. (PDF) [file pone.0288009.s004.pdf]

P. gingivalis

Palmitate

431

71

302

154

539

58

413

P. gingivalis+Palmitate

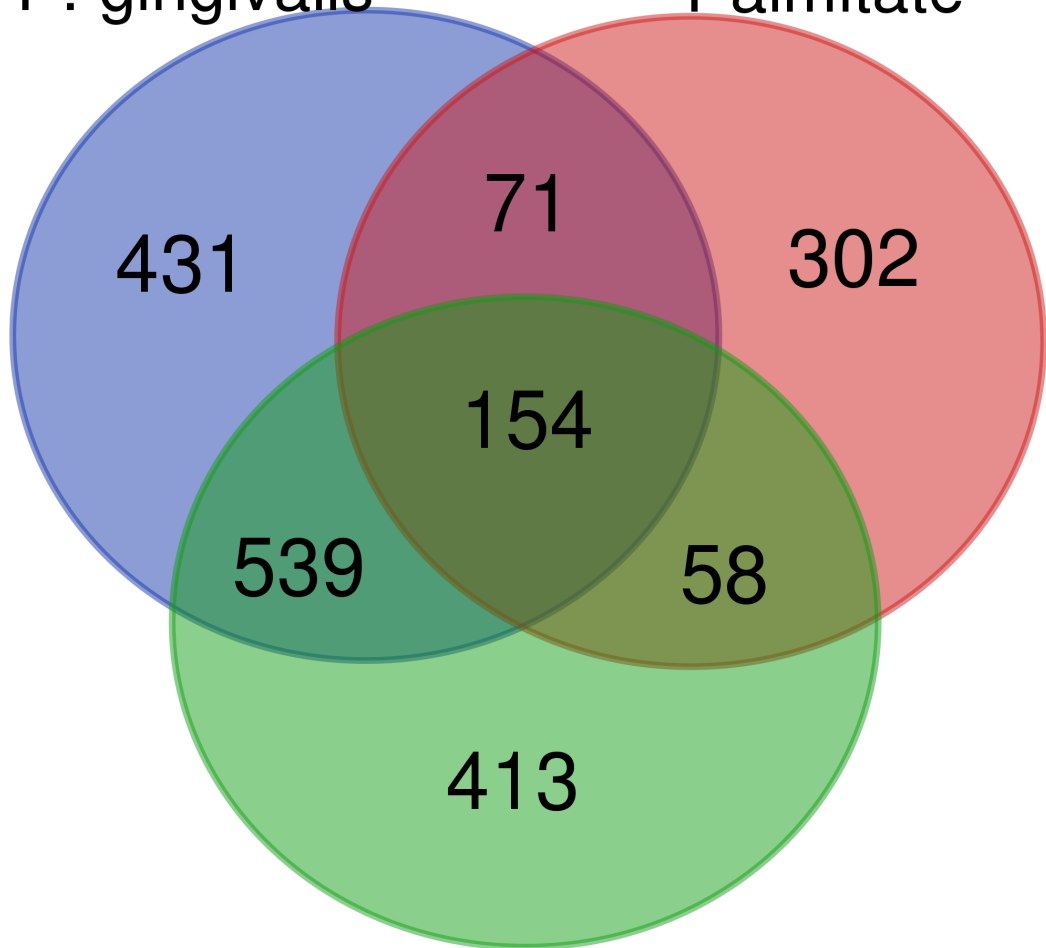

Supplement: S3 Fig — Microarray analysis was performed using the Affymetrix Clariom S Pico Array. 1.8-fold upregulated and downregulated genes were clustered with the Affymetrix Transcription Analysis Console (TAC) software (v.4.0.2.15). The Venn diagram was generated with the Bioinformatics & Evolutionary Genomics online tool from the University of Gent (available online at https://bioinformatics.psb.ugent.be/webtools/Venn). Each array was performed in duplicate. (PDF) [file pone.0288009.s005.pdf]

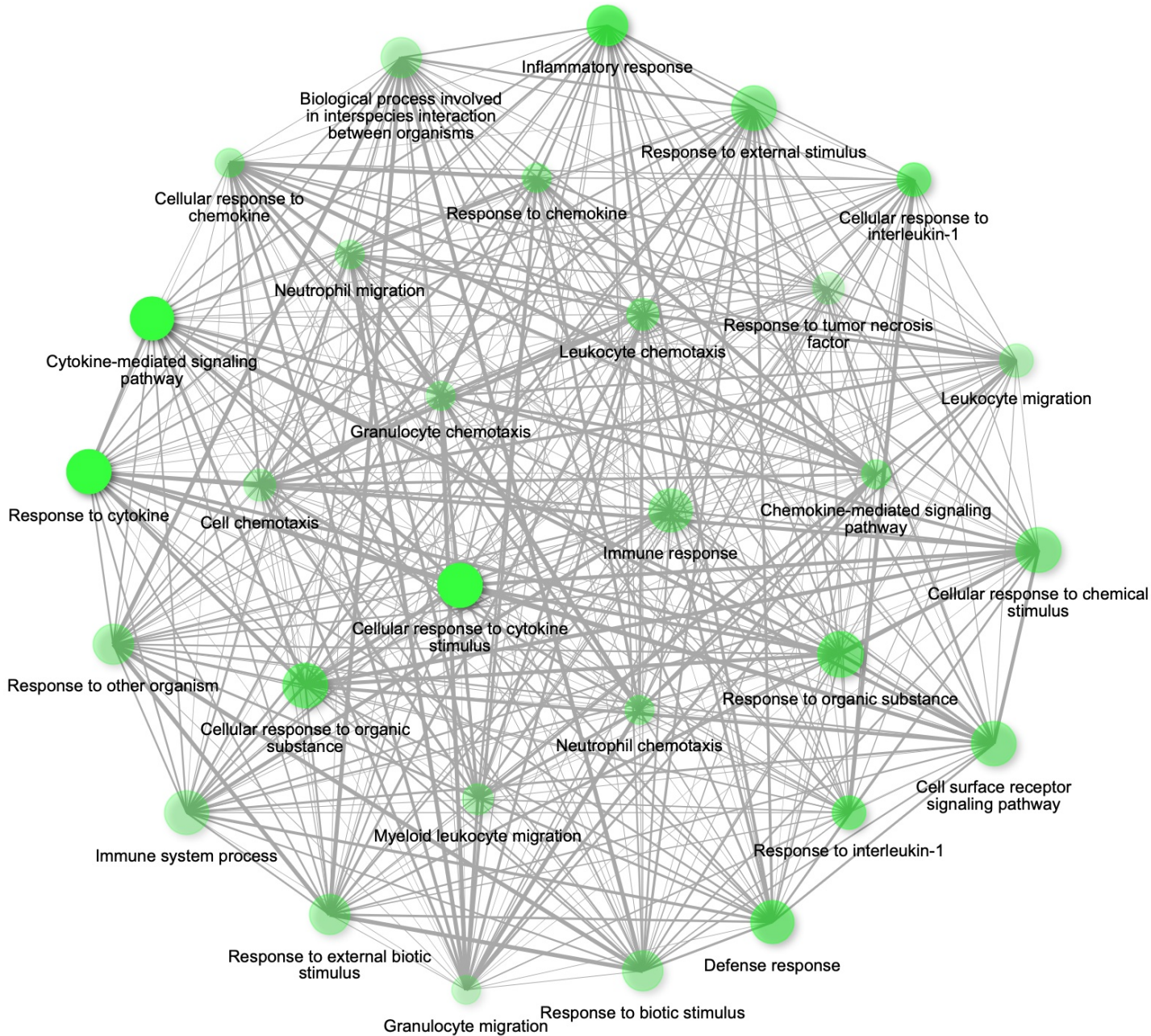

Supplement: S4 Fig — The gene ontology analysis was performed using ShinyGo version 0.75 (http://bioinformatics.sdstate.edu/go/) that was accessed online on March 11th, 2022. Bigger nodes in bright green represent enriched biological processes while smaller nodes in opaque green represent smaller gene sets and less enriched biological processes, respectively. (PDF) [file pone.0288009.s006.pdf]
